# Psychometric properties of health-related quality of life instruments used in survivors of critical illness: a systematic review

**DOI:** 10.1007/s11136-023-03487-x

**Published:** 2023-08-02

**Authors:** Sheraya De Silva, Nicholas Chan, Katherine Esposito, Alisa M. Higgins, Carol L. Hodgson

**Affiliations:** 1 Australian and New Zealand Intensive Care Research Centre (ANZIC-RC), School of Public Health and Preventive Medicine, Monash University, Melbourne, Australia; 2 Alfred Health, Melbourne, Australia

**Keywords:** Health-related quality of life, Critical care, Critical illness, Psychometric properties, Outcome assessment, Systematic review

## Abstract

**Background and objectives:**

Health-related quality of life (HRQoL) is a patient-reported measure of health status. However, research on the psychometric properties of HRQoL instruments used post-critical care is less common. We conducted a systematic review assessing the psychometric properties of HRQoL instruments used in adult survivors following critical illness.

**Methods:**

Three databases were systematically searched between 1990 and June 2022. Screening articles for eligibility, we selected either development studies for new tools or studies that evaluated psychometric properties, and whose target population represented adult survivors following critical illness. Methodological quality was assessed using the COnsensus-Based Standards for the selection of health Measurement INstruments (COSMIN) checklist. The results of each psychometric property were then assessed for criteria of good psychometric properties (sufficient, insufficient or indeterminate) and qualitatively summarised. Finally, we graded the quality of the evidence using a modified GRADE approach.

**Results:**

We retrieved 13 eligible studies from 2,983 records identifying 10 HRQoL instruments used post-critical illness. While high-quality evidence for the considered PROMs was limited primarily due to risk of bias, seven instruments demonstrated sufficient levels of reliability, four instruments presented sufficient hypothesis testing, and two instruments showed sufficient responsiveness. Except the Short Form-36, evidence for psychometric properties of other individual measures was limited to a few studies.

**Conclusion:**

There was limited evidence demonstrated for the psychometric properties of the included PROMs evaluating HRQoL. Further research is warranted to evaluate the psychometric properties of HRQoL measures, strengthening the evidence for administering these instruments in survivors following critical illness.

**Supplementary Information:**

The online version contains supplementary material available at 10.1007/s11136-023-03487-x.

## Plain English Summary

Health-related quality of life (HRQoL) is commonly measured in critical care research. However, there is currently no consensus on which instrument is most suitable to measure HRQoL in survivors following critical illness. In this systematic review, we assessed and compared reliability, validity and other measurement properties of HRQoL instruments. Our results found that almost all instruments demonstrated one or more measurement properties that supported its use. However, these tools require further evaluation before they should be routinely used for survivors of critical illness.

## Introduction

There has been a remarkable improvement in the survival of critically ill adult patients in the past two decades [1]. Hence, there is growing interest to explore and investigate long-term patient-reported outcome measures (PROMs) in survivors of critical illness, including health-related quality of life (HRQoL) [2].

HRQoL can be defined as a multidimensional construct that encapsulates physical health, mental health, and social functioning self-reported by an individual [3]**.** Several instruments have been developed, both generic and disease-specific, to evaluate HRQoL across different populations. In the context of intensive care, it may guide decision-making for the effective treatment choices for patients and their families to aid in recovery and resource allocation [4, 5]. However, there is no consensus on which instrument is the most suitable following critical illness. As HRQoL is a widely used outcome measure following critical illness and long-term, it is imperative to investigate the psychometric properties of each instrument to ensure reproducible, reliable results. Moreover, there must be a greater understanding of how relevant, comprehensive and comprehensible the items of each instrument are so that patients and/or proxies may report their physical and mental health as validly as possible. This information will also be essential in facilitating comparisons between different HRQoL instruments in this setting.

To this end, we conducted a systematic review to compare and examine the psychometric properties of HRQoL instruments administered post-discharge in adult survivors following critical illness.

## Methods

The protocol of this review was registered with PROSPERO (CRD42022340132), and it was completed in accordance with the Preferred Reporting Items for Systematic Reviews and Meta-Analyses (PRISMA) statement [6]. In June 2022, a systematic search was conducted on MEDLINE, EMBASE, and CINAHL to identify studies that evaluated psychometric properties of HRQoL instruments used post-critical care.

The search strategies were created with a combination of keywords (found in previous literature) and subject headings surrounding critical care, reliability, validity, responsiveness and minimal clinically important difference (MCID). We adapted the highly sensitive search filter developed by the COnsensus-based Standards for the selection of health Measurement Instruments (COSMIN) group in our search to identify relevant studies on psychometric properties [7]. There were no date restrictions in our search strategy. The full search strategies used in this review are outlined in Additional File: Table A1.

### Selection of studies for evidence

Two reviewers (SD and (NC or KE)) independently screened titles and abstracts of search results for eligibility using Covidence systematic review software (Veritas Health Innovation, Melbourne, Australia), followed by full-text review. Screening and full-text review conflicts were resolved by a third reviewer (AH or CH). Studies that represented adult survivors of critical illness (both immediately following ICU discharge and long-term follow-up) and assessed psychometric properties of new or existing HRQoL instruments were included in the review.

Exclusion criteria included studies whose samples predominantly did not represent the critical illness population or had a paediatric population, articles which did not report original data, studies that only measured HRQoL as an outcome without assessing psychometric properties, and publications not in the English language.

### Data extraction, psychometric property assessment and methodological risk of bias quality assessment

Data, extracted by two independent reviewers (SD and (NC or KE)), included bibliographic information, target population, sample size, characteristics of the HRQoL instruments, timepoint(s) that HRQoL data were collected and results for each psychometric property.

Definitions of each psychometric property are outlined in Additional Table A2. For the purpose of this study, the most critical psychometric properties are content validity and internal consistency [8].

The psychometric properties for each measurement tool within included studies was rated against COSMIN updated quality criteria for good psychometric properties and classified as sufficient ( +), insufficient (−) or indeterminate (?) (Table 1) [8]. With reference to hypothesis testing for construct validity, the review team formulated a set of hypotheses based on previous literature and included articles (Additional File: Table A3)*.*

**Table 1:** COSMIN updated criteria for good measurement properties. Mokkink, L.B., Prinsen, C.A.C., Patrick, D.L., Alonso, J., Bouter, L.M., de Vet, H.C.W., Terwee, C.B. (2018)

Measurement Property	Rating^1^	Criteria
Internal consistency	+	At least low evidence^2^ for structural validity^3^ AND Cronbach’s alpha(s) ≥ 0.70 for each unidimensional scale or subscale^4^
	?	Criteria for “At least low evidence^2^ for sufficient structural validity^3^” not met
	−	At least low evidence^2^ for structural validity^3^ AND Cronbach’s alpha(s) < 0.70 for each unidimensional scale or subscale^4^
Reliability	+	ICC or weighted Kappa ≥ 0.70
	?	ICC or weighted Kappa not reported
	−	ICC or weighted Kappa < 0.70
Hypothesis testing for construct validity	+	The result is in accordance with the hypothesis^5^
	?	No hypothesis defined (by the review team)
	−	The result is not in accordance with the hypothesis^5^
Responsiveness	+	The result is in accordance with the hypothesis^5^ OR AUC ≥ 0.70
	?	No hypothesis defined (by the review team)
	−	The result is not in accordance with the hypothesis^5^ OR AUC < 0.70

COSMIN methodology for systematic reviews of Patient-Reported Outcome Measures (PROMs): user manual. Version 1.0 [12]

*AUC* area under the curve

^1^ “ + ” = sufficient, “?” = indeterminate, “-” = insufficient

^2^As defined by grading the evidence using the GRADE approach

^3^This evidence may come from different studies

^4^The criteria “Cronbach’s alpha < 0.95” was deleted, as this is the development phase of a PROM and not when evaluating an existing PROM

^5^The results of all studies should be taken together and it should then be decided if 75% of the results are in accordance with the hypothesis. Hypothesis testing hypothesis: correlation coefficients ≥ 0.50

The methodological quality of included studies was critically appraised by two reviewers (SD and (NC or KE)) independently (with a third reviewer (AH or CH) resolving conflicts) using the COSMIN Risk of Bias checklist [9]. The tool utilises a four-point rating—“very good”, “adequate”, “doubtful” and “inadequate”. It comprises ten boxes with standards referring to design requirements and statistical methods for evaluating the methodological quality of single studies. Each box provides an overall rating for PROM development, content validity, structural validity, internal consistency, cross-cultural validity, reliability, measurement error, criterion validity, hypothesis testing for construct validity and responsiveness. The overall score for each psychometric property was determined by taking the lowest rating of any standard in the box.

To determine content validity and PROM Development, the relevance, comprehensibility and comprehensiveness of the PROM is evaluated. However, PROM development assesses newly developed instruments while content validity assesses existing instruments [10].

When assessing methodological quality of existing PROMs, reviewers were instructed to check if PROM development ratings for these instruments were available in a table published on the COSMIN website. If this was the case, the reviewers independently entered these existing ratings to our review accordingly [11].

### Summary of findings and grading of the quality of evidence

The findings of each instrument in the included studies per psychometric property were qualitatively summarised, accompanied with an overall rating of sufficient ( +), insufficient (− ), inconsistent ( ±) or indeterminate (?) [8]. If results were found to be inconsistent, we checked if a majority of the results were either sufficient or insufficient and rated accordingly [8]. If this was not the case, the results remained as inconsistent. Two independent reviewers (SD and (NC or KE)) then graded the quality of the evidence as either high, moderate, low or very low using a modified Grading of Recommendations Assessments, Development and Evaluation (GRADE) approach. Quality of evidence is downgraded if there is risk of bias, inconsistency, imprecision and/or indirectness. If there was risk of bias, downgrading was categorised as either serious, very serious or extremely serious risk of bias [8, 12]. More detailed information downgrading based on these factors are available in Additional File: Tables A4 and A5. For results that were inconsistent or indeterminate, quality of the evidence was not graded [8]. Any discrepancies were resolved by a third reviewer (AH or CH).

### Formulating recommendations

The results of this review were used to formulate recommendations on suitable PROMs [8]. In order to arrive at such a recommendation, the included PROMs were sorted into three categories:

1. PROMs with evidence for sufficient content validity (at any level), and at least low-quality evidence for sufficient internal consistency.
2. PROMs with high-quality evidence for an insufficient psychometric property.
3. PROMs categorised in neither 1 nor 2.

If PROMs were categorised in 1, they were recommended for use. If they were categorised in 2, they were not recommended for use. If PROMs were categorised in 3, they were noted as measures potential for use but requiring further evaluation.

## Results

### Search results

**Fig. 1:**
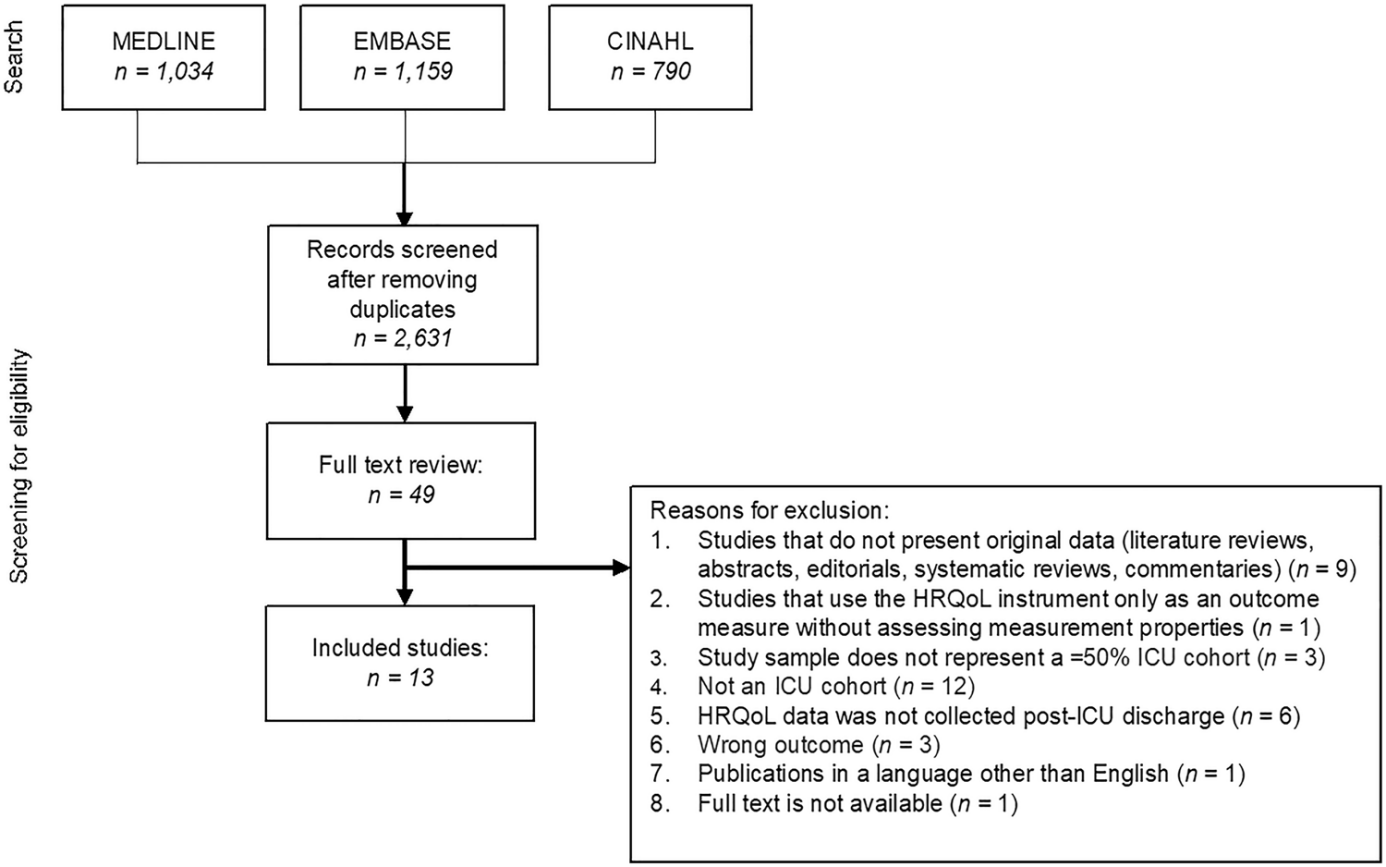
Flowchart of study selection

All considered PROMs and characteristics of the included studies are detailed in Table 2 and Additional Tables A6-A8. We screened 2983 studies for eligibility, of which 352 duplicates were discarded. The titles and abstracts of 2631 articles were screened for eligibility which yielded 49 articles for full-text review. Of these, 13 articles, which evaluated ten HRQoL questionnaires, were eligible for inclusion in this review (Fig. 1).

**Table 2:** Characteristics of the included studies

Author (Year)	Country	HRQoL instrument					
		Instrument Used	Mode of Administration	Number of items	Timepoint(s) that HRQoL data were collected	Target Population	Sample Size
Capuzzo (2000)	Italy	QOL-IT and QOL-SP	Direct interview or Telephone interview *Reported by patient only*	QOL-IT: 5 QOL-SP: 15	Prior to ICU admission and 12 months after ICU discharge	General ICU	172
Chrispin (1997)	UK	SF-36 (UK version)	Self-Administration *Reported by patient only*	36	At ICU discharge	General ICU	166
Fernandez (1996)	Spain	QOL-SP	Direct interview or Telephone interview *Reported by patient and/or proxy*	15	Prior to ICU admission and 6 months after ICU discharge	General ICU	578
Heyland (2000)	Canada	SF-36	Telephone interview *Reported by patient only*	36	Prior to ICU admission and mean 16.6 months after hospital discharge	Sepsis	30
Jones (1993)	UK	Whiston Health Questionnaire	Self-Administration *Reported by patient only*	21	Prior to ICU admission, 6 months after ICU discharge and 12 months after ICU discharge	General ICU	85
Kaarlola (2004)	Finland	EQ-5D-3L (Finnish translation) and RAND-36 (Finnish translation)	Self-Administration *Reported by patient only*	EQ-5D-3L: 6 RAND-36: 36	Prior to ICU admission and 12 months to 72 months after ICU discharge	General ICU	1099
Kawakami (2021)	Japan	SF-36 (Japanese translation)	Self-Administration *Reported by patient and/or proxy*	36	Prior to ICU admission and 6 months after ICU admission	General ICU	96
Khoudri (2007)	Morocco	SF-36 (Arabic translation)	Direct interview or Telephone interview *Reported by patient only*	36	3 months after ICU discharge	Medical ICU	145
Lipsett (2000)	USA	SIP and MSF-36	Direct interview, Telephone interview or Self-Administration *Reported by patient and/or proxy*	SIP: 136 MSF-36: 20	Prior to ICU admission, 1 month, 3 months, 6 months and 12 months after ICU discharge	Surgical ICU	127
Malmgren (2021)	Sweden	Provisional questionnaire on HRQoL post-intensive care	Self-Administration *Reported by patient only*	238	6 months to 36 months after ICU discharge	General ICU	395
McNelly (2016)	UK	SF-36 (UK version)	Direct interview *Reported by patient only*	36	18 months after ICU discharge	General ICU	27
Rogers (1997)	UK	SF-36 (UK version, slightly modified to accommodate the relatives’ perspective of the patients’ health)	Self-Administration *Reported by patient and/or proxy*	36	Prior to ICU admission and 6 months after ICU discharge	General ICU	99
Skinner (2013)	Australia	AQoL and SF-6D	Direct interview or Telephone interview *Reported by patient only*	AQOL: 15 SF-6D: 11	As soon as participants were able after ICU admission and 6 months after ICU discharge	General ICU	67

*ICU* intensive care unit, *CCU* critical care unit, *HDU* high dependency unit, *AQoL* assessment of quality of life, *EQ-5D-3L* EuroQol 5-dimension 3-level, *HRQoL* health-related quality of life, *SF-36* short form-36, *SF-6D* short form-6 dimension, *SIP* sickness impact profile, *MSF-36* modified short form-36, *QOL-IT* Italian quality of life questionnaire, *QOL-SP* Spanish quality of life questionnaire

At least one psychometric property was reported in each of the eligible studies. Of the ten instruments, eight were generic (EuroQol 5-dimension 3-level (EQ-5D-3L), Assessment of Quality of Life (AQoL), Short Form-36 (SF-36), Short Form-6D (SF-6D), Modified Short Form-36 (MSF-36), Sickness Impact Profile (SIP), Spanish Quality of Life Questionnaire (QOL-SP) and Italian Quality of Life Questionnaire (QOL-IT)), while two were developed specifically for critically ill patients (Whiston Health Questionnaire, and the provisional questionnaire developed by Malmgren et al.) [4, 5, 13–23]. Of the included articles, 3 (23%) were development studies of new HRQoL tools while 10 (77%) studies investigated the psychometric properties of existing HRQoL instruments. Of the 13 studies, 6 (46%) articles were comparative studies between two or more instruments whereas 7 (54%) studies individually assessed the psychometric properties of one instrument only. The SF-36 was administered across seven studies, while the QOL-SP was used in two studies. The other instruments were evaluated in only one study.

Of the included studies, 7 (54%) administered the questionnaires as an interview to survivors of critical illness while in 7 (54%) studies, survivors self-administered the tools. Among the 6 (46%) studies that solely conducted interviews for HRQoL, 4 (67%) used both direct and telephone interviews while 1 (17%) used only direct interviews and 1 (17%) used telephone interviews. Two (13%) studies had mixed modes of administration. Nine (60%) studies collected HRQoL data prior to ICU admission and survivors were followed up post-ICU discharge. Follow-up assessments for HRQoL data collection occurred between 1 and 72 months post-discharge among our included studies with 6 or 12 months being the most common timepoints. Twelve (80%) studies measured HRQoL as a long-term outcome while one (7%) study reported HRQoL at ICU discharge. The majority of the HRQoL instruments were administered by researchers with clinical experience and experience in qualitative research or nursing staff trained in using and administering the instruments.

Target populations of all included studies were from the general ICU, conducted in the USA (1 (8%)), UK (4 (31%)), Italy (1 (8%)), Sweden (1 (8%)), Finland (1 (8%)), Japan (1 (8%)), Spain (1 (8%)), Canada (1(8%)), Morocco (1 (8%)) and Australia (1 (8%)). with sample sizes ranging from 27 to 1,099. While 10 (77%) studies used the original questionnaires, Japanese, Arabic and Finnish translations of the SF-36 were used as well as an EQ-5D-3L instrument translated in Finnish. Among the 13 studies, 9 (69%) were conducted over 15 years ago while 4 (31%) studies were published since 2010.

Psychometric property assessment is reported in Table 3, while methodological quality is presented in Table 4. A summary of findings and quality of evidence is detailed in Table 5.

**Table 3:** Results of the measurement properties and quality criteria rating

HRQoL instrument	Author (Year)	Country	Psychometric Properties [Sample size (n) and rating (+ /?/−)]					
			Reliability	Internal Consistency	Hypothesis testing for construct validity	Content Validity	Responsiveness	MCID
EQ-5D-3L	Kaarlola (2004)	Finland	N/E	N/E	*n* = 1,099 (?)	*n* = 1,099	N/E	N/E
SF-36	Chrispin (1997)	UK	*n* = 166 ( +)	*n* = 166 (?)	*n* = 166 ( +)	*n* = 166	N/E	N/E
	Heyland (2000)	Canada	n = 26 (−)	*n* = 26 (?)	*n* = 30 ( +)	N/E	N/E	N/E
	Kaarlola (2004)	Finland	N/E	N/E	*n* = 1,099 (?)	N/E	N/E	N/E
	Kawakami (2021)	Japan	N/E	N/E	N/E	*n* = 93	*n*-96 ( +)	*n* = 96
	Khoudri (2007)	Morocco	*n* = 73 ( +)	*n* = 145 (?)	*n* = 145 ( +)	N/E	N/E	N/E
	McNelly (2016)	UK	N/E	N/E	*n* = 27 (−)	*n* = 27	N/E	N/E
	Rogers (1997)	UK	*n* = 99 ( +)	*n* = 99 (?)	*n* = 99 ( +)	N/E	N/E	N/E
MSF-36	Lipsett (2000)	USA	*n* = 10 ( +)	*n* = 127 (?)	*n* = 127 (?)	*n* = 127	N/E	N/E
SIP	Lipsett (2000)	USA	*n* = 10 ( +)	*n* = 127 (?)	*n* = 127 (?)	*n* = 127	N/E	N/E
AQoL	Skinner (2013)	Australia	*n* = 67 ( +)	*n* = 67 (?)	N/E	N/E	*n* = 67 (−)	N/E
SF-6D	Skinner (2013)	Australia	*n* = 67 ( +)	*n* = 67 (?)	N/E	N/E	*n* = 67 (−)	N/E
QOL-SP	Capuzzo (2000)	Italy	*n* = 36 ( +)	*n* = 36 (?)	*n* = 172 ( +)	N/E	N/E	N/E
	Fernandez (1996)	Spain	N/E	*n* = 578 (?)	*n* = 578 ( +)	N/E	*n* = 578 ( +)	N/E
QOL-IT	Capuzzo (2000)	Italy	*n* = 36 ( +)	*n* = 36 (?)	*n* = 172 ( +)	N/E	N/E	N/E
Provisional questionnaire	Malmgren (2021)	Sweden	N/E	N/E	N/E	*n* = 395	N/E	N/E
Whiston Health Questionnaire	Jones (1993)	UK	N/E	N/E	*n* = 49 (6-month follow-up), 42 (12-month follow-up) ( +)	N/E	N/E	N/E

Quality Criteria Ratings: ( +) = sufficient, (?) = indeterminate, (−) = insufficient [rated in all measurement properties except content validity and MCID]

*AQoL* assessment of quality of life, *EQ-5D-3L* EuroQol 5-dimension 3-level, *HRQoL* health-related quality of life, *SF-36* short form-36, *SF-6D* short form-6 dimension, *MSF-36* modified short form-36, *SIP* sickness impact profile, *QOL-IT* Italian quality of life questionnaire, *QOL-SP* Spanish quality of life questionnaire, *MCID* minimal clinically important difference

N/E = not evaluated

**Table 4:** Methodological quality of the included studies

HRQoL instrument	Author (Year)	PROM Development	Content Validity	Internal Consistency	Reliability	Hypothesis Testing	Responsiveness
EQ-5D-3L	Kaarlola (2004)	Inadequate	N/E	N/E	N/E	Adequate	N/E
SF-36	Chrispin (1997)	Inadequate	N/E	Very good	Inadequate	Adequate	N/E
	Heyland (2000)	Inadequate	N/E	Very good	Adequate	Very good	N/E
	Kaarlola (2004)	Inadequate	N/E	N/E	N/E	Adequate	N/E
	Kawakami (2021)	Inadequate	N/E	N/E	N/E	N/E	Very good
	Khoudri (2007)	Inadequate	N/E	Very good	Very good	Very good	N/E
	McNelly (2016)	Inadequate	N/E	N/E	N/E	Inadequate	N/E
	Rogers (1997)	Inadequate	N/E	Very good	Very good	Very good	N/E
MSF-36	Lipsett (2000)	Inadequate	N/E	Very good	Inadequate	Doubtful	N/E
SIP	Lipsett (2000)	Inadequate	N/E	Very good	Inadequate	Doubtful	N/E
SF-6D	Skinner (2013)	Inadequate	N/E	Inadequate	Inadequate	N/E	Inadequate
AQoL	Skinner (2013)	Inadequate	N/E	Inadequate	Inadequate	N/E	Inadequate
QOL-SP	Capuzzo (2000)	Inadequate	N/E	Inadequate	Adequate	Doubtful	N/E
	Fernandez (1996)	Inadequate	N/E	Very good	Inadequate	Inadequate	Inadequate
QOL-IT	Capuzzo (2000)	Very good	N/E	Inadequate	Adequate	Doubtful	N/E
Provisional questionnaire	Malmgren (2021)	Adequate	N/E	N/E	N/E	N/E	N/E
Whiston Health Questionnaire	Jones (1993)	Inadequate	N/E	N/E	N/E	Inadequate	N/E

N/E = not evaluated

*AQoL* assessment of quality of life, *EQ-5D-3L* EuroQol 5-dimension 3-level, *HRQoL* health-related quality of life, *SF-36* short form-36, *SF-6D* short form-6 dimension, *MSF-36* modified short form-36, *QOL-IT* Italian quality of life questionnaire, *QOL-SP* Spanish quality of life questionnaire

**Table 5:** Summary of findings and grading the quality of evidence for each measurement property

**Internal Consistency**	**Overall Rating**	**Total Sample size**	**Quality of Evidence**
EQ-5D-3L	N/E	N/E	N/E
SF-36	?	436	N/E
MSF-36	?	127	N/E
SIP	?	127	N/E
SF-6D	?	67	N/E
AQoL	?	67	N/E
QOL-SP	?	614	N/E
QOL-IT	?	36	N/E
Provisional questionnaire	N/E	N/E	N/E
Whiston Health Questionnaire	N/E	N/E	N/E
**Reliability**	**Overall Rating**	**Total Sample Size**	**Quality of Evidence**
EQ-5D-3L	N/E	N/E	N/E
SF-36	+	N/E	Low
MSF-36	+	10	Very low
SIP	+	10	Very low
SF-6D	+	67	Very low
AQoL	+	67	Very low
QOL-SP	+	36	Very low
QOL-IT	+	36	Very low
Provisional questionnaire	N/E	N/E	N/E
Whiston Health Questionnaire	N/E	N/E	N/E
**Hypotheses testing**	**Overall Rating**	**Total Sample Size**	**Quality of Evidence**
EQ-5D-3L	?	1,099	N/E
SF-36	+	1,566	Low
MSF-36	?	127	N/E
SIP	?	127	N/E
SF-6D	N/E	N/E	N/E
AQoL	N/E	N/E	N/E
QOL-SP	+	750	Low
QOL-IT	+	172	Low
Provisional questionnaire	N/E	N/E	N/E
Whiston Health Questionnaire	+	42	Very low
**Responsiveness**	**Overall Rating**	**Total Sample Size**	**Quality of Evidence**
EQ-5D-3L	N/E	N/E	N/E
SF-36	+	96	Moderate
MSF-36	N/E	N/E	N/E
SIP	N/E	N/E	N/E
SF-6D	−	67	Very low
AQoL	−	67	Very low
QOL-SP	+	578	Very low
QOL-IT	N/E	N/E	N/E
Provisional questionnaire	N/E	N/E	N/E
Whiston Health Questionnaire	N/E	N/E	N/E

Overall ratings: ( +) = sufficient, (− ) = insufficient, ( ±) = inconsistent, (?) = indeterminate, N/E = not evaluated

*AQoL* assessment of quality of life, *EQ-5D-3L* = EuroQol 5-dimension 3-level, *HRQoL* health-related quality of life, *SF-36* short form-36, *SF-6D* short form-6 dimension, *MSF-36* modified short form-36, *QOL-IT* Italian quality of life questionnaire, *QOL-SP* Spanish quality of life questionnaire

### Short form-36 (SF-36)

The SF-36 is a 36-item generic questionnaire comprising 2 composite scores (physical and mental composites), measuring 8 dimensions of health [24]. The SF-36 version 2 was the most commonly used instrument in 6 of 13 (46%) studies, while the RAND-36-item health survey (based on SF-36 version 1) was used in one study (7.7%). Internal consistency, reliability, hypothesis testing for construct validity and responsiveness of the SF-36 were reported [5, 13–18].

Content validity was reported in four studies [13–15, 17]; however, assessment was not conducted as the definition of content validity did not coincide with COSMIN’s interpretation. These studies observed the distribution of scores across domains and reported any floor or ceiling effects.

The quality of evidence of its internal consistency across 4 studies was not graded – it was considered indeterminate due to no evidence of structural validity [5, 13, 16, 18].

Reliability from 4 studies was considered sufficient, and quality of evidence was downgraded due to risk of bias [5, 13, 16, 18]. With reference to our team’s hypotheses for construct validity, the pooled result was sufficient. One study that reported convergent validity (between SF-36 and Patrick’s Perceived Quality of Life) reported sufficient results [5]. One study comparing the SF-36 against other physical activity measures did not adhere to any hypothesis, thereby rendered insufficient [17]. The convergent validity between the EQ-5D-3L and the RAND-36 in one study was considered indeterminate [14]. Despite the authors stating that the associations between domain and composite scores of the RAND-36 and EQ-5D-3L presented strong correlations, the data of these correlation coefficients could not be found in the publication [14]. The results in all 3 studies investigating known-groups validity were sufficient [13, 16, 18]. We downgraded the quality of evidence of pooled sufficient hypothesis testing for construct validity by two levels to low due to very serious risk of bias.

Responsiveness was examined in one study and rated as sufficient, with moderate-quality evidence [4].

### EuroQol 5-dimension 3-level (EQ-5D-3L)

The EQ-5D-3L comprises a descriptive system (with five dimensions of health) and a visual analogue scale that rates an individual’s health between 0 and 100 [25]. Preference weights are applied for each answer in the descriptive system, generating utility scores which are used to derive quality-adjusted life years (QALYs).

Construct validity, on the basis of convergent validity in one study, was considered indeterminate as correlation coefficients for associations between domain scores and composite scores of the EQ-5D-3L and RAND-36 were not reported [14].

### Modified short form-36 (MSF-36)

The MSF-36 is a 20-item survey adapted from the SF-36 with 6 dimensions of health determined most important by patients [19]. The MSF-36 was assessed for its internal consistency, reliability and construct validity in only one study, in conjunction with the SIP [19].

Content validity was reported; however, assessment was not conducted as the definition of content validity did not coincide with COSMIN’s interpretation—the authors investigated the distribution of the domain scores.

Internal consistency was indeterminate. Reliability, on the other hand, was sufficient. Reliability of the MSF-36 had very low-quality evidence as the study was of inadequate quality, downgrading the quality of evidence by three levels (extremely serious risk of bias).

Hypothesis testing for construct validity, on the basis of known-groups validity, was rated indeterminate as correlation coefficients for the MSF-36 in relation to gender and age 1 year following critical illness were absent.

### Sickness impact profile (SIP)

The SIP is a 136-item multidimensional instrument containing 12 dimensions [26]. In conjunction with the MSF-36, the SIP was assessed in one study for its reliability, internal consistency and construct validity [19].

Content validity, assessed as the distribution of domain scores, was not examined for the SIP. Internal consistency was indeterminate due to no evidence for sufficient structural validity. Reliability, on the contrary, was sufficient but there was very low-quality evidence due to extremely serious risk of bias. Hypothesis testing, on the basis of known-groups validity, was considered indeterminate as there were no correlation coefficients of the SIP with age and gender reported.

### Short form-6D (SF-6D)

Based on the SF-36, the SF-6D comprises six dimensions and eleven items from the SF-36 [27]. Preference weights are applied for each answer, deriving utility scores which are thereby used to generate QALYs. One study compared the SF-6D and AQoL for their internal consistency, reliability and responsiveness [4].

Internal consistency was indeterminate. Reliability of the SF-6D was sufficient and it had insufficient responsiveness as the effect sizes for changes in scores pre-ICU and post-ICU scores were below 0.50. The quality of evidence for reliability and responsiveness of the SF-6D was very low due to inadequate study quality (extremely serious risk of bias).

### Assessment of quality of life version 1 (AQoL)

The AQoL is a 15-item questionnaire comprising 5 dimensions [28]. Just like the SF-6D, preference weights are applied for each answer to derive utility scores, used to generate QALYs. As above, the AQoL was compared against the SF-6D for its internal consistency, reliability and responsiveness [4].

Internal consistency was indeterminate, while reliability of the AQoL was sufficient. Responsiveness of the AQoL was rated insufficient as the effect sizes in changes in scores pre-ICU and post-ICU were lower than 0.50. The quality of evidence for its reliability and responsiveness was very low due to extremely serious risk of bias.

### Spanish quality of life questionnaire (QOL-SP)

Designed specifically for critically ill patients, the QOL-SP is a 15-item questionnaire and categorised into three subscales [20]. The QOL-SP was administered in 2 studies [20, 21]. Reliability, internal consistency, construct validity and responsiveness (assessed in one study only) were evaluated in the QOL-SP [20, 21].

QOL-SP had sufficient reliability, hypothesis testing and responsiveness. The pooled result for internal consistency was indeterminate. Reliability had a very low quality of evidence as one study had adequate quality and the other had inadequate study quality. Additionally, the sample size for evaluating reliability was low. Hypothesis testing had a low quality of evidence as the two studies were doubtful and inadequate quality, respectively, hence very serious risk of bias. Quality of evidence for responsiveness was very low due to inadequate study quality (extremely serious risk of bias).

### Italian quality of life questionnaire (QOL-IT)

The QOL-IT, adapted from the QOL-SP, comprises 5 items and it is administered to critically ill patients [21]. The study that used the QOL-IT investigated its internal consistency, reliability and construct validity [21]. Sufficient reliability and hypothesis testing were found while internal consistency was considered indeterminate.

Very low-quality evidence for reliability was due to two reasons. It was downgraded by one level as only one adequate quality study was available, and by two levels due to a small sample size. Hypothesis testing had low-quality evidence due to doubtful study quality (very serious risk of bias).

### Provisional questionnaire

The provisional questionnaire by Malmgren et al., a 238-item questionnaire measuring long-term HRQoL and burden of disease following critical illness, was administered to survivors between 6 and 36 months after intensive care [23]. The study reviewed its development by assessing relevance, comprehensiveness and comprehensibility. Methodological quality and grading were not conducted for this instrument as no other psychometric properties were assessed.

### Whiston health questionnaire

Developed by Jones et al., the Whiston Health Questionnaire (WHQ) was administered to survivors 6 months and 12 months following critical illness [22]. It measures change in health status in adult survivors before and after critical care, containing 21 items. Hypothesis testing for construct validity between the WHQ, Functional Limitations Profile and Perceived Quality of Life scale was sufficient, and its quality of evidence was very low due to inadequate study quality (extremely serious risk of bias).

## Discussion

Among 2983 records, our review retrieved 13 studies evaluating 10 HRQoL instruments. The results indicate that 7 instruments (SF-36, MSF-36, SIP, SF-6D, AQoL, QOL-IT, QOL-SP) demonstrated sufficient reliability, while 4 instruments (SF-36, QOL-SP, QOL-IT, Whiston Health Questionnaire) presented sufficient hypotheses testing for construct validity, 2 instruments (SF-36, QOL-SP) had sufficient responsiveness and none of the instruments had sufficient internal consistency. None of the PROMs presented high-quality evidence for any measurement property largely due to poor methodological quality. Methodological quality depends on components within each psychometric property, detailed below.

Intraclass correlation coefficients (ICCs) were used in most instruments, resulting in sufficient reliability. The ICC is considered preferential for reliability statistics as it accounts for systematic errors between repeated measurements [29]. With reference to hypotheses testing, our set of hypotheses allowed us to evaluate the magnitude of construct validity between two instruments or subgroups without relying on merely statistical significance. None of the included PROMs had sufficient internal consistency due to no evidence of structural validity, which is a mandatory requirement.

Other features in our review included the ability to identify newer, disease-specific HRQoL measures such as the provisional questionnaire by Malmgren et al. [23]. Both generic and disease-specific instruments are essential in clinical research and policy analysis [30]. The SF-36 is a generic instrument routinely used in critical care research, and it was the most commonly used instrument in our review [31]. Generic instruments have been essential for comparing different interventions, informed healthcare resource allocation and policy-making for such interventions across different populations [30]. However, disease-specific instruments are also necessary to identify the specific concerns of the patient with a certain condition and for measuring small, clinically important changes [30].

Two previous systematic reviews by Robinson and colleagues, and Black et al., similarly aimed to assess the psychometric properties of HRQoL measures in adult intensive care survivors (but also included non-ICU patients such as high dependency unit patients) [32, 33]. Our results build on existing evidence of the review by Robinson and colleagues, wherein 47% of their eligible studies were also included in our review [32]. While the majority of our results are in line with their findings, there are a few key differences in our review that may provide a clearer interpretation. Firstly, for instruments reported by more than one study, we pooled our results to allow for an overall sufficient, insufficient or indeterminate or inconsistent rating for a psychometric property. On the contrary, Robinson and colleagues reported each psychometric property for each instrument separately for each study. Unlike Robinson’s study, we decided to evaluate instruments used in more than one study. We also graded the quality of the evidence to ascertain how trustworthy our results were, which was not conducted in Robinson’s review. Another systematic review by Black and colleagues assessed the SF-36 and SIP [33]. Similarly, they found sufficient reliability in the two measures. However, contrary to insufficient responsiveness of the SF-36 and SIP in our results, Black and colleagues reported sufficient responsiveness in these measures. It is important to mention that information on responsiveness of these measures was limited, therefore the authors sought information on responsiveness of the SF-36 in studies that included patients beyond critical care. Black and colleagues also did not grade the quality of the evidence. In contrast to these two previous systematic reviews, we restricted our target population to only patients from the ICU. Lastly, our review assessed MCID as observing the smallest change in HRQoL in each individual patient aids in clinical, patient-centred decision-making over the course of a disease [34]. This was reported in one of the studies in our review, and its relevance and importance warrant further research [15].

Based on our key findings, we could not recommend a suitable instrument for use. This is primarily due to content validity, which is considered the most important psychometric property, and internal consistency [10]. The COSMIN initiative recommends that evidence for sufficient content validity and at least low-level evidence for sufficient internal consistency are mandatory to consider them suitable for use [8]. Generating sufficient internal consistency requires evidence for sufficient structural validity as mentioned above. On the other hand, we had difficulty evaluating content validity although it was reported in 54% of our included studies. We did not assess content validity where it was reported in studies which did not address the relevance, comprehensiveness or comprehensibility of a questionnaire [10]. Most included studies in this review assessed content validity based on the distribution of scores. Secondly, one study reported content validity of a new PROM under development before substantial adjustments were made to the final PROM [10, 23]. Therefore, it was considered for PROM development instead, which examines the same elements as content validity, except on new PROMs (while content validity is assessed on existing PROMs). Addressing content validity is essential to identify irrelevant, missing items in a questionnaire that could potentially limit other psychometric properties such as reliability and internal consistency [35]. Our review seldom found studies wherein survivors following critical illness or proxies were interviewed on which concepts in the questionnaires were relevant to their health, easy to understand and if any items were missing. Development of the provisional HRQoL instrument by Malmgren et al. was an ideal example of how content validity is assessed in accordance with the COSMIN framework [23]. The authors conducted cognitive interviews on survivors following critical illness, field notes were taken for better understanding of issues, meetings were recorded, and interviews followed a semi-structured guideline. Therefore, this study was able to examine the relevance, comprehensiveness and comprehensibility of the questionnaire during its development.

None of the instruments demonstrated high-quality evidence for any measurement property. The COSMIN group states that any PROMs with high-quality evidence for insufficient psychometric properties should not be recommended for use [8]. Although some measurement properties of included PROMs were insufficient, the quality of evidence was either low or very low due to risk of bias. Hence, none of the included PROMs fell under this category.

With increased importance of HRQoL post-critical care today, very few systematic reviews have investigated the research quality of the instruments used [32, 33]. Taking our results and COSMIN’s guidelines into consideration, all PROMs evaluated in this review have the potential to be recommended but they must undergo further evaluation [8]. Future validation studies are necessary as most instruments are newly developed and/or reported in only one study, not all psychometric properties were evaluated per instrument, and most validation studies in this review were published over 15 years ago. We recommend that psychometric properties are assessed in conjunction with COSMIN’s methodology. Therefore, adequate statistical methods, and appropriate definitions per psychometric property, could yield sufficient results. Additionally, adhering to COSMIN’s guidelines will reduce the risk of bias which is a major contributing factor to the poor quality of evidence. Incorporating such guidelines in the future may potentially aid in selecting an appropriate HRQoL PROM.

Other avenues for future research include thorough assessment of content validity, structural validity and internal consistency. Furthermore, conducting comparative studies on the psychometric properties of generic vs disease-specific instruments in a post-critical care setting is desirable. Lastly, HRQoL has been considered in multiple core outcome sets (COS) in critical care survivorship as of 2020 including patients with post-intensive care syndrome, physical rehabilitation, extracorporeal membrane oxygenation and intermittent mandatory ventilation [36]. If future evaluation of disease-specific HRQoL instruments is evidently of high research quality, there is potential to establish recommendations for instruments in COS in the critical care setting. Likewise, adequate psychometric properties of the SF-36 which is commonly used in critical care will strengthen its role in the existing core outcome measurement sets.

Our review had limitations which must be acknowledged. Our search strategy was limited to only English articles; hence, non-English articles with key findings may have been excluded. Moreover, we adapted the search filter from COSMIN to retrieve relevant articles—however, its sensitivity may have reduced making it more likely to miss articles applicable to our entry criteria. Five psychometric properties were not evaluated as they were not investigated in the included studies. Strengths in our review include following the COSMIN guidelines, which are universally accepted in selecting suitable, psychometrically sound PROMs. Furthermore, our inclusion criteria focussed on only HRQoL of people post-critical care, making indirectness less likely to occur.

## Conclusion

This systematic review identified numerous HRQoL instruments, both generic and disease-specific, available for administration after critical illness. We found that seven instruments had sufficient reliability (SF-36, MSF-36, SIP, SF-6D, AQoL, QOL-IT, QOL-SP), four had sufficient hypotheses testing (SF-36, QOL-SP, QOL-IT, Whiston Health Questionnaire), and two had sufficient responsiveness (SF-36, QOL-SP). No PROM reported high-quality evidence for any measurement property. Conforming to COSMIN guidelines, there was limited evidence demonstrated for the psychometric properties of all included PROMs. Further research is warranted to evaluate psychometric properties of PROMs used post-critical care using COSMIN methodology. This will strengthen the evidence for administering HRQoL instruments on survivors following critical illness.

### Supplementary Information

Below is the link to the electronic supplementary material.

Supplementary file1 (DOCX 43 KB)
