# Supplementary material for: Psychometric properties of health-related quality of life instruments used in survivors of critical illness: a systematic review
Source: Qual Life Res. 2023 Aug 2;33(1):17–29. doi: 10.1007/s11136-023-03487-x (PMC10784337; doi:10.1007/s11136-023-03487-x)
Supplement: Supplementary file 1 — Supplementary file1 (DOCX 43 KB) [file 11136_2023_3487_MOESM1_ESM.docx]

**Article Title:** Psychometric properties of health-related quality of life instruments used in survivors of critical illness: a systematic review

**Journal Name:** Quality of Life Research

Sheraya De Silva^1^, Nicholas Chan^1^, Katherine Esposito^2^, Alisa M. Higgins^1*^, Carol L. Hodgson^1*^

1. Australian and New Zealand Intensive Care Research Centre (ANZIC-RC), School of Public Health and Preventive Medicine, Monash University, Melbourne, Australia
2. Alfred Health, Melbourne, Australia

*Share equal responsibilities as senior authors for this review

Corresponding Author:

Sheraya De Silva

Australian and New Zealand Intensive Care Research Centre, School of Public Health and Preventive Medicine, Monash University, Australia

[wadu.desilva@monash.edu](mailto:wadu.desilva@monash.edu)

# Additional File

| Table A1: Electronic database search strategy.   \| MEDLINE \| 1 \| (clinimetr* OR clinometr*).ti,ab OR reproducib*.mp OR reliab*.mp OR valid*.mp OR unreliab*.mp OR responsive*.mp OR interpretab*.mp OR “minimal* adj clinical* difference”.mp OR “minimum adj clinical* adj important difference”.mp OR “minimal* important difference” OR “minimum important difference”.mp OR “internal consistency”.ti,ab OR “(measurement OR psychometric) adj propert*”.ti,ab,kw \| \| --- \| --- \| --- \| \| 2 \| “health status indicators”/ OR “validation study”/ OR “comparative study”/ OR psychometrics/ OR “observer variation”/ OR “reproducibility of results”/ OR “discriminant analysis”/ OR “minimally clinically important difference”/ \| \| 3 \| 1 OR 2 \| \| 4 \| *”quality of life”/ \| \| 5 \| (“health-related quality of life” OR “health related quality of life” OR “quality-of-life” OR “quality of life” OR “life quality” OR HRQOL OR HRQL OR HQOL OR HQL OR QL OR QOL).ti,ab,kw \| \| 6 \| (“short form-36” OR “short form 36” OR “36-item short form” OR “SF-36” or “SF-12” or “short form-12” OR “12-item short form” OR “EuroQol” OR “EQ-5D” or “EuroQol 5-dimension” OR “EQ-5D-3L” OR “EQ-5D-5L” OR “EuroQol 5-dimension 3-level” OR “EuroQol 5-dimension 5-level” OR “Assessment of Quality of Life” or “AQoL” or “Health Utilities Index” or “HUI”).mp \| \| 7 \| OR/4-6 \| \| 8 \| (((critical OR intensive) adj2 (treatment* OR care)) OR (critical* adj2 (ill* OR patient*)) OR ICU OR ITU OR CCU).mp \| \| 9 \| “critical illness”/ OR “critical care”/ OR “intensive care units”/ \| \| 10 \| 8 OR 9 \| \| 11 \| 3 AND 7 AND 10 \| \| 12 \| Limit 11 to (“all infant (birth to 23 months)” OR “all child (0 to 18 years)” OR “newborn infant (birth to 1 month)” OR “infant (1 to 23 months)” OR “preschool child (2 to 5 years)” OR “child (6 to 12 years)” OR “adolescent (13 to 18 years)”) \| \| 13 \| 12 NOT 13 \| \|  \| \| \| \| EMBASE \| 1 \| (clinimetr* OR clinometr*).ti,ab OR reproducib*.mp OR reliab*.mp OR valid*.mp OR unreliab*.mp OR responsive*.mp OR interpretab*.mp OR “minimal* adj clinical* difference”.mp OR “minimum adj clinical* adj important difference”.mp OR “minimal* important difference” OR “minimum important difference”.mp OR “internal consistency”.ti,ab OR “(measurement OR psychometric) adj propert*”.ti,ab,kw \| \| 2 \| “health status indicator”/ OR “validation study”/ OR “comparative study”/ OR psychometry/ OR “observer variation”/ OR reproducibility/ OR “discriminant analysis”/ OR “minimally clinically important difference”/ \| \| 3 \| Reliability/ OR validity/ OR “construct validity”/ OR “criterion related validity”/ OR “face validity”/ OR “content validity”/ OR “internal consistency”/ OR “measurement error”/ \| \| 4 \| OR/1-3 \| \| 5 \| *”quality of life”/ \| \| 6 \| (“health-related quality of life” OR “health related quality of life” OR “quality-of-life” OR “quality of life” OR “life quality” OR HRQOL OR HRQL OR HQOL OR HQL OR QL OR QOL).ti,ab,kw \| \| 7 \| (“short form-36” OR “short form 36” OR “36-item short form” OR “SF-36” or “SF-12” or “short form-12” OR “12-item short form” OR “EuroQol” OR “EQ-5D” or “EuroQol 5-dimension” OR “EQ-5D-3L” OR “EQ-5D-5L” OR “EuroQol 5-dimension 3-level” OR “EuroQol 5-dimension 5-level” OR “Assessment of Quality of Life” or “AQoL” or “Health Utilities Index” or “HUI”).mp \| \| 8 \| OR/5-7 \| \| 9 \| (((critical OR intensive) adj2 (treatment* OR care)) OR (critical* adj2 (ill* OR patient*)) OR ICU OR ITU OR CCU).mp \| \| 10 \| “critical illness”/ OR “intensive care”/ OR “critically ill patient”/ \| \| 11 \| 9 OR 10 \| \| 12 \| 4 AND 8 AND 11 \| \| 13 \| Limit 12 to (embryo <first trimester> or infant <to one year> or child <unspecified age> or preschool child <1 to 6 years> or school child <7 to 12 years> or adolescent <13 to 17 years>) \| \| 14 \| 12 NOT 13 \| \|  \| \| \| \| CINAHL \| 1 \| ( TI "(measurement OR psychometric) propert*" OR AB "(measurement OR psychometric) propert*") OR TX "(measurement OR psychometric) propert*")) OR (TI clinimetr* OR AB clinimetr*) OR (TI clinometr* OR AB clinometr*) OR reproducib* OR reliab* OR valid* OR unreliab* OR responsive* OR interpretabl* OR "minimal* clinical* important difference" OR "minimum clinical* important difference" OR "minimal* important difference" OR "minimum important difference" OR (TI "internal consistency" OR AB "internal consistency") \| \| 2 \| (MH "Health Status Indicators") OR (MH "validation studies") OR (MH "comparative studies") OR (MH "psychometrics) OR (MH "reproducibility of results") OR (MH "discriminant analysis") \| \| 3 \| (MH "reliability and validity") OR (MH "reliability") OR (MH "internal consistency") OR (MH "measurement error") OR (MH "validity") OR (MH "criterion-related validity") OR (MH "content validity") OR (MH "construct validity") OR (MH "face validity") \| \| 4 \| S1 OR S2 OR S3 \| \| 5 \| (MM "quality of life") \| \| 6 \| (TI "health-related quality of life" OR AB "health-related quality of life" OR TX "health-related quality of life") OR (TI "health related quality of life" OR AB "health related quality of life" OR TX "health related quality of life") OR (TI "quality-of-life" OR AB "quality-of-life" OR TX "quality-of-life") OR (TI "quality of life" OR AB "quality of life" OR TX "quality of life") OR (TI "life quality" OR AB "life quality" OR TX "life quality") OR "HRQOL" OR "HRQL" OR "HQOL" OR "HQL" OR "QL" OR "QOL" \| \| 7 \| "short form-36" OR "short form 36" OR "36-item short form" OR "SF-36" or "SF-12" OR "short form-12" OR "short form 12" OR "12-item short form" OR "EuroQol" or "EQ-5D" OR "EuroQol 5-dimension" OR "EQ-5D-3L" OR "EQ-5D-5L" OR "EuroQol 5-dimension-3-level" OR "EuroQol 5-dimension-5-level" OR "Assessment of quality of life" OR "AQoL" OR "Health Utilities Index" OR "HUI" \| \| 8 \| S5 OR S6 OR S7 \| \| 9 \| (((critical OR intensive) N2 (treatment* OR care)) OR (critical* N2 (ill* OR patient*)) OR ICU OR ITU OR CCU) \| \| 10 \| (MH "critical illness") OR (MH "critically ill patients") OR (MH "intensive care units") \| \| 11 \| S9 OR S10 \| \| 12 \| S4 AND S8 AND S11 \| \| 13 \| S4 AND S8 AND S11  **Limiters** - Age Groups: Fetus, Conception to Birth, Infant, Newborn 0-1 month, Infant, 1-23 months, Child, Preschool 2-5 years, Child, 6-12 years, Adolescence, 13-18 years, All Infant, All Child \| \| 14 \| S12 NOT S13 \| |
| --- | --- | --- | --- | --- | --- | --- | --- | --- | --- | --- | --- | --- | --- | --- | --- | --- | --- | --- | --- | --- | --- | --- | --- | --- | --- | --- | --- | --- | --- | --- | --- | --- | --- | --- | --- | --- | --- | --- | --- | --- | --- | --- | --- | --- | --- | --- | --- | --- | --- | --- | --- | --- | --- | --- | --- | --- | --- | --- | --- | --- | --- | --- | --- | --- | --- | --- | --- | --- | --- | --- | --- | --- | --- | --- | --- | --- | --- | --- | --- | --- | --- | --- | --- | --- | --- | --- | --- | --- | --- | --- | --- |

**Table A2:** Definitions of measurement properties. Adapted from Mokkink, L.B., Prinsen, C.A.C., Patrick, D.L., Alonso, J., Bouter, L.M., de Vet, H.C.W., Terwee, C.B. (2018). COSMIN methodology for systematic reviews of Patient-Reported Outcome Measures (PROMs): user manual. Version 1.0.

| Measurement property | Definition |
| --- | --- |
| Reliability | Assesses if a PROM produces consistent results. |
| Internal consistency | Assesses if there are correlations between items within the same PROM. |
| Content validity | Assesses if a PROM can represent HRQoL. |
| Hypotheses testing for construct validity. | Assesses if the scores of a PROM adhere to hypotheses set by the review team (either between the PROM and another comparator instrument or between subgroups) |
| 1. Convergent validity | Comparison of the PROM with other outcome comparator instruments. |
| 1. Known-groups validity | Comparison of the PROM scores between subgroups. |
| Responsiveness | Assesses if the PROM can detect any changes in HRQoL over time. |
| Structural Validity | Assesses if the scores of a PROM are representative of the PROM’s dimensionality. |
| Abbreviations: HRQoL = health-related quality of life, PROM = patient-reported outcome measure. | |
|  | |

**Table A3:** Set of hypotheses to test for construct validity and responsiveness.

| 1 | The correlation coefficients between the investigated PROM and the comparator instrument both measuring HRQoL is 0.50 or more.^1^ |
| --- | --- |
| 2 | The changes observed between subgroups using the same PROM are clinically meaningful. **^1^** |
| 3 | The correlation coefficients between the investigated HRQoL PROM and the comparator instrument measuring a different construct is less than 0.30. **^1^** |
| 4 | At least 50% of the correlation coefficients between the domains of the investigated HRQoL PROM and the comparator instrument measuring a different construct is less than 0.30. **^1^** |
| 5 | At least 50% of the correlation coefficients between the domains of the investigated PROM and the comparator instrument both measuring HRQoL is 0.50 or more. **^1^** |
| 6 | Effect sizes of the investigated PROM and the comparator instrument both measuring the same construct is 0.50 or more. **^2^** |
| 7 | Effect sizes of two composite scores within the investigated PROM is 0.50 or more.^2^ |
| 8 | AUC is 0.70 or more.^2^ |
| 9 | Kappa index between the investigated PROM and comparator instrument both measuring the same construct is 0.50 or more.^2^ |
| ^1^Construct validity, ^2^Responsiveness  Abbreviations: PROM = Patient-Reported Outcome Measure, HRQoL = Health-Related Quality of Life, AUC = Area under the curve | |

**Table A4:** Definitions of quality levels using the modified GRADE approach. Adapted from Mokkink, L.B., Prinsen, C.A.C., Patrick, D.L., Alonso, J., Bouter, L.M., de Vet, H.C.W., Terwee, C.B. (2018). COSMIN methodology for systematic reviews of Patient-Reported Outcome Measures (PROMs): user manual. Version 1.0.

| Quality Level | Definition |
| --- | --- |
| High | There are no concerns about the quality of the evidence. We are very confident that our summarized result of the measurement property is trustworthy. |
| Moderate | We are moderately confident that our pooled result of the measurement property is trustworthy. |
| Low | We have limited confidence that our summarized result of the measurement property is trustworthy. |
| Very Low | We have very little confidence that the summarized result of the measurement property is trustworthy. |
| These definitions were adapted from the GRADE approach. | |

**Table A5:** Factors of the GRADE approach. Mokkink, L.B., Prinsen, C.A.C., Patrick, D.L., Alonso, J., Bouter, L.M., de Vet, H.C.W., Terwee, C.B. (2018). COSMIN methodology for systematic reviews of Patient-Reported Outcome Measures (PROMs): user manual. Version 1.0.

| Factors | Description |
| --- | --- |
| Risk of bias | Doubtful or inadequate quality of the included studies |
| Inconsistency | If inconsistency was only due to results rated + and ?, then the quality of the evidence was downgraded by one. If inconsistency comprised a mix of + and –, then we decided not to grade the quality of the evidence* |
| Imprecision | Downgraded by one if the total sample size was between 50-100, downgraded by two if the total sample size was below 50. |
| Indirectness | Downgraded by one if the HRQoL construct was administered partly to the target population* |
| *decided by the review team. | |

**Table A6:** Domains of HRQoL instruments. Mokkink, L.B., Prinsen, C.A.C., Patrick, D.L., Alonso, J., Bouter, L.M., de Vet, H.C.W., Terwee, C.B. (2018). COSMIN methodology for systematic reviews of Patient-Reported Outcome Measures (PROMs): user manual. Version 1.0.

| HRQoL instrument | Domains/Dimensions |
| --- | --- |
| EQ-5D-3L | 5 dimensions: Mobility, Personal Care, Usual Activities, Pain/Discomfort and Anxiety/Depression. Visual Analogue Scale along 0-100 (0 = worst imaginable health state, 100 = best imaginable health state). |
| SF-36 | 8 domains (Physical Functioning, Role Physical, Bodily Pain, General Health, Vitality, Social Functioning, Role Emotional, Mental Health). |
| MSF-36 | 6 domains (Physical, Social, Role Functioning, Mental Health, Health Perception, Bodily Pain). |
| SIP | 12 domains (Ambulation, Mobility, Body Care, Social Interactions, Alertness Behaviour, Emotional Behaviour, Communication, Sleep and Rest, Eating, Work, Home Management, Recreation) |
| SF-6D | 6 domains (Physical Functioning, Role Limitations, Social Functioning, Pain, Mental Health, and Vitality) |
| AQoL | 5 domains (Illness, Independent Living, Social Relationships, Physical Sense, Psychological Wellbeing |
| QOL-SP | 3 domains (Basic Physiological Activiies, Normal Daily Activities, and Emotional State) |
| QOL-IT | 5 domains (Physical Activity, Social Life, Perceived Quality of LIFE, Oral Communication, and Functional Limitation) |
| Provisional questionnaire | 13 domains (Cognitive Function, Fatigue, Physical Health, Pain, Psychological Aspects, Activities of Daily Living, Sleep, Appetite and Alcohol, Sexual Health, Sensory Functions, Gastrointestinal Functions, Urinary Functions, and Work Life) |
| Whiston Health Questionnaire | Not reported |
| Abbreviations: AQoL = Assessment of Quality of Life, EQ-5D-3L = EuroQol 5-dimension 3-level, EQ-5D-5L = EuroQol 5-dimension 5-level, HRQoL = health-related quality of life, SF-36 = short form-36, SF-6D = short form-6 dimension, MSF-36 = modified short form-36, QOL-IT = Italian Quality of Life Questionnaire, QOL-SP = Spanish Quality of Life Questionnaire. | |

**Table A7:** Summary of results of included studies. Adapted from: Mokkink, L.B., Prinsen, C.A.C., Patrick, D.L., Alonso, J., Bouter, L.M., de Vet, H.C.W., Terwee, C.B. (2018). COSMIN methodology for systematic reviews of Patient-Reported Outcome Measures (PROMs): user manual. Version 1.0.

| Internal Consistency | Summary of results |  |  |
| --- | --- | --- | --- |
| EQ-5D-3L | N/E |  |  |
| SF-36 | Summarised Cronbach alpha = 0.64-0.99  Total sample size = 436 | |  |
| MSF-36 | Summarised Cronbach alpha = 0.92-0.95  Total sample size = 127 |  |  |
| SIP | Summarised Cronbach alpha = 0.35-0.93  Total sample size = 127 |  |  |
| SF-6D | Summarised Cronbach alpha = 0.65  Total sample size = 67 |  |  |
| AQoL | Summarised Cronbach alpha = 0.81  Total sample size = 67 |  |  |
| QOL-SP | Summarised Cronbach alpha = 0.81-0.85  Total sample size = 614 |  |  |
| QOL-IT | Summarised Cronbach alpha = 0.76  Total sample size = 36 |  |  |
| Provisional questionnaire | N/E |  |  |
| Whiston Health Questionnaire | N/E |  |  |
|  | | | |
| Reliability | **Summary of results** |  |  |
| EQ-5D-3L | N/E |  |  |
| SF-36 | ICC range = 0.75-0.97  Sample size = 364 |  |  |
| MSF-36 | ICC range = > 0.90  Sample size = 10 |  |  |
| SIP | ICC range = > 0.90  Sample size = 10 |  |  |
| SF-6D | ICC range = 0.71  Sample size = 67 |  |  |
| AQoL | ICC range = 0.71  Sample size = 67 |  |  |
| QOL-SP | ICC range = > 0.90  Sample size = 36 |  |  |
| QOL-IT | ICC range = > 0.90  Sample size = 36 |  |  |
| Provisional questionnaire | N/E |  |  |
| Whiston Health Questionnaire | N/E |  |  |
|  |  |  |  |
| Hypotheses testing | **Summary of results** |  |  |
| EQ-5D-3L | No hypotheses confirmed. |  |  |
| SF-36 | 2 hypotheses confirmed. |  |  |
| MSF-36 | No hypotheses confirmed. |  |  |
| SIP | No hypotheses confirmed. |  |  |
| SF-6D | N/E |  |  |
| AQoL | N/E |  |  |
| QOL-SP | 1 hypothesis confirmed. |  |  |
| QOL-IT | 1 hypothesis confirmed. |  |  |
| Provisional questionnaire | N/E |  |  |
| Whiston Health Questionnaire | 1 hypothesis confirmed. |  |  |
|  |  |  |  |
| Responsiveness | **Summary of results** |  |  |
| EQ-5D-3L | No hypotheses confirmed. |  |  |
| SF-36 | 1 hypothesis confirmed. |  |  |
| MSF-36 | N/E |  |  |
| SIP | N/E |  |  |
| SF-6D | No hypotheses confirmed. |  |  |
| AQoL | No hypotheses confirmed. |  |  |
| QOL-SP | 1 hypothesis confirmed. |  |  |
| QOL-IT | N/E |  |  |
| Provisional questionnaire | N/E |  |  |
| Whiston Health Questionnaire | N/E |  |  |
| Overall ratings: (+) = sufficient, (-) = insufficient, (±) = inconsistent, (?) = indeterminate, N/E = not evaluated  Abbreviations: AQoL = Assessment of Quality of Life, EQ-5D-3L = EuroQol 5-dimension 3-level, EQ-5D-5L = EuroQol 5-dimension 5-level, HRQoL = health-related quality of life, SF-36 = short form-36, SF-6D = short form-6 dimension, MSF-36 = modified short form-36, QOL-IT = Italian Quality of Life Questionnaire, QOL-SP = Spanish Quality of Life Questionnaire. | |  |  |

**Table A8:** Results for measurement properties of instruments.

|  | Psychometric Properties [Sample size (n), result and rating (+/?/-)] | | | | | |
| --- | --- | --- | --- | --- | --- | --- |
|  | Reliability | **Internal Consistency** | **Hypothesis testing for construct validity** | **Content Validity** | **Responsiveness** | **MCID** |
| EQ-5D-5L | | | | | | |
| Kaarlola (2004) | N/E | N/E | Individual and summary elements of the EQ-5D-3L and SF-36 correlated strongly with the two-tailed Spearman’s p. | Ceiling effect was detected with the EQ-5D-3L. | N/E | N/E |
| SF-36 |  |  |  |  |  |  |
| Chrispin (1997) | Reliability coefficient ≥ 0.75 (all domains) | Cronbach’s alpha ≥ 0.70 (all domains) | Significant difference in distribution of scores due to age, sex and interaction between age and sex. All mean scores for women were lower than men except for the Role Emotional domain. Mean total scores generally declined with age for both sexes, although a peak in score is observed in the 25-34 age-group for both sexes. | n=166  Broad distribution of scores observed in the Physical Functioning, Bodily Pain, General Health, Vitality, and Social Functioning domains. Mental Health domain showed an approximately normal distribution. Highly skewed distribution for Role Emotional and Role Physical domains. | N/E | N/E |
| Heyland (2000) | ICC ≥ 0.70 (all domains except Physical Functioning, General Health, Vitality, and Social Functioning) | Cronbach’s alpha ≥ 0.7 (all domains except General Health) | Pearson’s correlation coefficient 0.56 and 0.45 indicating significant correlations between MCS and PQOL scores. | N/E | N/E | N/E |
| Kaarlola (2004) | N/E | N/E | Individual and summary elements of the EQ-5D-3L and RAND-36 correlated strongly with the two-tailed Spearman’s p. | N/E | N/E | N/E |
| Kawakami (2021) | N/E | N/E | N/E | Floor effect of PCS score at baseline and 6 months was 4.3% and 3.2% respectively.  Ceiling effect of PCS score and floor and ceiling effect of MCS score were not observed. | Effect size between Global Assessment Rating negative change and change in PCS and MCS scores was 0.72 and 0.50. Moderate responsiveness between the change in SF-36 PCS and MCS scores and negative change in Global Assessment Rating. AUC (PCS) = 0.69, AUC (MCS) = 0.64 | PCS score: 6.5, MCS score: 8.0 |
| Khoudri (2007) | ICC ≥ 0.70 (all domains) | Cronbach’s alpha ≥ 0.70 (all domains) | Age and sex: significant decline in scores with age except for the Bodily Pain and Mental Health domains.  Lower scores were reported by women in all domains except Social Functioning and Role Emotional domains.  Burden of chronic illness: decline in all domains except Bodily Pain, Role Emotional, and Mental Health domains.  Education: Higher levels of education indicated higher SF-36 scores in all domains except Bodily Pain and Role Emotional.  Marital Status: single survivors reported better scores and differences were found in all scales except Role Emotional and Mental Health. | N/E | N/E | N/E |
| McNelly (2016) | N/E | N/E | Significant correlations of SF-36 PCS, MCS and PF scores with CFS and steps/d. | 0% floor effect was seen across cohorts and domains, though 11.1% of healthy patients rated PF at maximal scores. | N/E | N/E |
| Rogers (1997) | At discharge: reliability coefficients ≥ 0.70 (all domains).  6 months follow-up: reliability coefficients ≥ 0.70 (all domains). | Cronbach’s alpha ≥ 0.70 (all domains except Role Emotional at discharge) | Discriminant validity at discharge: correlation coefficients for the patients’ and relatives’ assessments of the same domain are high, with values greater than 0.60 in all domains except Role Emotional and Mental Health.  Discriminant validity at the 6 month follow-up: correlation coefficients for assessments of the same domain were greater than at discharge, with values greater than 0.70 in all domains except Mental Health. | N/E | N/E | N/E |
| MSF-36 |  |  |  |  |  |  |
| Lipsett (2000) | Correlation coefficients > 0.90 | Cronbach’s alpha ≥ 0.70 at baseline, 6 months, and 12 months only. | Baseline: women had significantly lower scores in the Activities and Health Perception domains.  After 1 year: No significant difference between men and women in any of the domains. | Baseline: The scores for each dimension were distributed throughout the entire range. The dimension score of health perception was skewed (0.87) toward poorer perception.  At 3 months and 6 months: for total scores, the data were significantly skewed. | N/E | N/E |
| SIP |  |  |  |  |  |  |
| Lipsett (2000) | Correlation coefficients > 0.90 | Cronbach’s alpha ≥ 0.70 at all timepoints. | N/E | SIP score in individual dimensions re skewed at different time points, only at 1 month was there a significantly skewed distribution of results (1.01) for the total SIP score. | N/E | N/E |
| AQoL |  |  |  |  |  |  |
| Skinner (2013) | ICC ≥ 0.70 (all domains) | Baseline: Cronbach’s alpha ≥ 0.70  Follow-up: Cronbach’s alpha ≥ 0.70 | N/E | N/E | Effect size = 0.13  6 months post-admission rehabilitation status RE: 1.00.  Increased requirement for community services RE: 1.00  6 months general health status RE: 1.00 | N/E |
| SF-6D |  |  |  |  |  |  |
| Skinner (2013) | ICC ≥ 0.70 (all domains) | Baseline: Cronbach’s alpha < 0.70  Follow-up: Cronbach’s alpha ≥ 0.70 | N/E | N/E | Effect size = 0.43  6 months post-admission rehabilitation status RE: 1.29  Increased requirement for community services RE: 0.20  6 months general health status RE: 0.60 | **N/E** |
| QOL-SP |  |  |  |  |  |  |
| Capuzzo (2000) | weighted kappa ≥ 0.70 | Cronbach’s alpha ≥ 0.70 | n=172  Validation according to functional limitation (absent, mild, dependent): statistically significant increase in QOL-SP median score (2 to 6 to 12). | N/E | N/E | N/E |
| Fernandez (1996) | N/E | Cronbach’s alpha ≥ 0.70 | Factorial analysis confirmed that the three subscales were fundamental questionnaire components – existence of three factors which together explained 59% of the total variability.  The first factor (related to the items of Normal Daily Activities subscale) explained 36.5%, the second factor (related to items of Emotional State subscale) explained 14.4% and the third factor (based on items of Basic Physiological subscale) explained 7.9%.  Convergent validity: significant concordance between QOL-SP and GOS results. | N/E | weighted kappa index = 0.56, p < 0.0001 | N/E |
| QOL-IT |  |  |  |  |  |  |
| Capuzzo (2000) | weighted kappa ≥ 0.70 | n=36  Cronbach’s alpha ≥ 0.70 | n=172  Validation according to functional limitation (absent, mild, dependent): statistically significant increase in QOL-IT median score (3 to 6 to 13). | N/E | N/E | N/E |
| Provisional Questionnaire |  |  |  |  |  |  |
| Malmgren (2021) | N/E | N/E | N/E | Evidence of content validity was demonstrated: this questionnaire was based mainly on issues reported by ICU survivors themselves, all interviewees read the field notes to ensure proper understanding of the term “issues,” cognitive interviews were conducted, and the researchers permitted participants to add potentially missing issues in the quantitative phase. | N/E | N/E |
| Whiston Health Questionnaire | | | | | | |
| Jones (1993) | N/E | N/E | Score obtained at follow-up correlated well with the scores provided by the FLP (r=0.70, p<0.0001) and PQOL (r=0.678, p<0.0001). | N/E | N/E | N/E |
